# The myeloid sarcoma treated by Venetoclax with hypomethylating agent followed by stem cell transplantation: rare case report

**DOI:** 10.1186/s12905-021-01328-y

**Published:** 2021-05-01

**Authors:** Aleksina Shatilova, Larisa Girshova, Daniil Zaytsev, Irina Budaeva, Yuliya Mirolyubova, Darya Ryzhkova, Roman Grozov, Konstantin Bogdanov, Tatiana Nikulina, Dmitriy Motorin, Darina Zammoeva, Svetlana Efremova, Vladimir Ivanov, Alexey Petukhov, Yuliya Alekseeva, Andrey Zaritskey

**Affiliations:** 1 Personalized Medicine Centre, Almazov National Medical Research Centre, 2 Akkuratova Str., Saint Petersburg, Russian Federation 197341; 2 Almazov National Medical Research Center, 2 Akkuratova Str., Saint Petersburg, Russian Federation 197341

**Keywords:** Myeloid sarcoma of the uterine cervix, Venetoclax, Hypomethylating agent, Stem cell transplantation, Acute myeloid leukemia

## Abstract

**Background:**

Myeloid sarcoma (MS) is a very rare condition, develops both in patients with other hematological neoplasms, and as isolated tumor. MS of the gynecologic tract is extremely rare. An available literature data about diagnosis and management of MS is summarized in the article. The role of chemotherapy, radiation therapy, surgery and bone marrow transplantation in the treatment is discussed. Polychemotherapy and allogeneic bone marrow transplantation were suggested to be the optimal treatment strategy of MS of the gynecological tract. The use of new targeted agents results in promising clinical data.

**Case presentation:**

We are presenting a rare clinical case of a MS of the uterine cervix with concomitant bone marrow involvement and describe all the peculiarities of the clinical course, diagnosis, and treatment. The patient received chemotherapy followed by allogeneic bone marrow transplantation. The pre-transplant therapy allowed us to perform allogeneic bone marrow transplantation with the deepest response possible: complete PET-negative and MRD-negative remission of the disease.

**Conclusions:**

MS remains a subject of discussion regarding its diagnostic and therapeutic aspects. The use of novel targeting agents can be perspective option for patient with extramedullary disease.

## Background

MS (also known as chloroma or granulocytic sarcoma) is a very rare condition characterized by proliferation of immature myeloid cells in extramedullary sites [1]. Chloroma most often develops in patients with acute myeloid leukemia (AML), other myeloproliferative neoplasms, or myelodysplastic syndromes, but it can be also presented as an isolated mass. MS, associated with AML, may precede it, develop during the onset or relapse of disease [2–4]. Untreated isolated MS usually leads to AML at a median of 7 months (range of 1 to 2) [5]. Although MS has been reported in various parts of the body, the most common sites are lymph nodes, soft tissues, and bones [5]. MS of the gynecologic tract, in particular the cervix, is rare (up to 5.8–22.7% of all localizations). Vaginal bleeding is a very common presenting symptom of MS of the cervix or uterus [6, 7]. Diagnosis of MS is difficult, especially in the absence of bone marrow involvement, and is based on a combination of clinical features, radiological investigations, and tissue biopsy [1, 8, 9]. Immunohistochemical analysis is an important part of MS diagnostics, which allows establishing the correct diagnosis in 96% of cases [5, 8]. FDG-PET/CT is a useful tool to estimate the extent of the lesion [10]. Treatment of MS with AML (Cytarabine-based) protocols (regardless of bone marrow involvement) is the most reasonable approach [1]. Successful use of targeted agents such as Gemtuzumab ozogamicin, hypomethylating agents (Decitabine and 5-azacitidine), and BCL-2 inhibitors (Venetoclax) has also been described [11–14]. Surgical and radiation therapies are accepted treatment methods, but their roles in the treatment algorithm are not well-defined. Surgical intervention should be considered before the systemic treatment in patients with symptoms of mass effect or when excision biopsy was necessary to establish the diagnosis [1]. Radiation is used in addition to chemotherapy in patients with isolated MS, who do not achieve complete regression of the tumor mass after polychemotherapy, as well as palliative option when compression symptoms (superior vena cava syndrome, spinal cord roots compression) are present [1, 11, 15, 16]. The hematopoietic stem cell transplantation, is an important therapeutic option which can be used in patients with and without bone marrow involvement [1, 8, 17]. In this article, we present a rare clinical case of an MS of the uterine cervix with concomitant bone marrow involvement.

## Case presentation

**Fig. 1:**
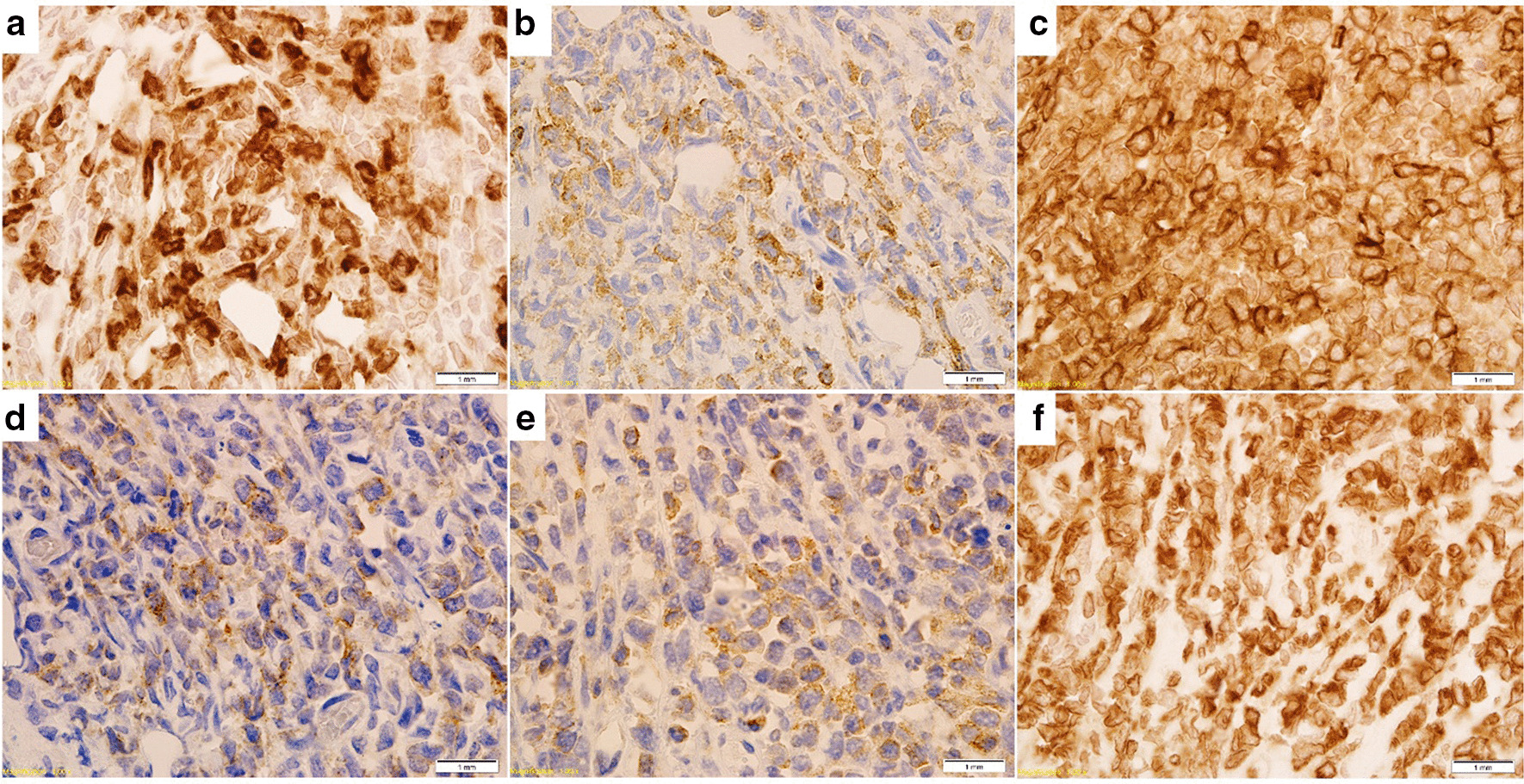
Immunohistochemical study of MS. **a** Expression of CD15; **b** expression of CD45; **c** expression of CD99; **d** expression of CD117; **e** expression of myeloperoxidase, **f** expression of BCL-2

A 49-year-old female with ECOG Performance Score of 0 presented with complaints of profuse metrorrhagia following period of amenorrhea. There was no history of weight loss, fever, or night sweats. A speculum examination showed an exophytic tumor originating from the uterine cervix with contact bleeding. The histological and immunohistochemical study of the biopsy showed that the biopsied tissue was diffuse infiltrated by polymorphous cells with positivity for MPO, LCA, CD99, CD4, CD117, CD15, BCL-2, and negativity for lymphoid, epithelial, and neuroendocrine markers (MCК, CК7, ALK, CD10, CD20, CD3, CD30, CD34, chromogranin A, synaptophysin, desmin, Fli-1, S-100, PAX-4, CD56, CD7) (Fig. 1). The Ki67 was expressed in 40% of the cells.

**Fig. 2:**
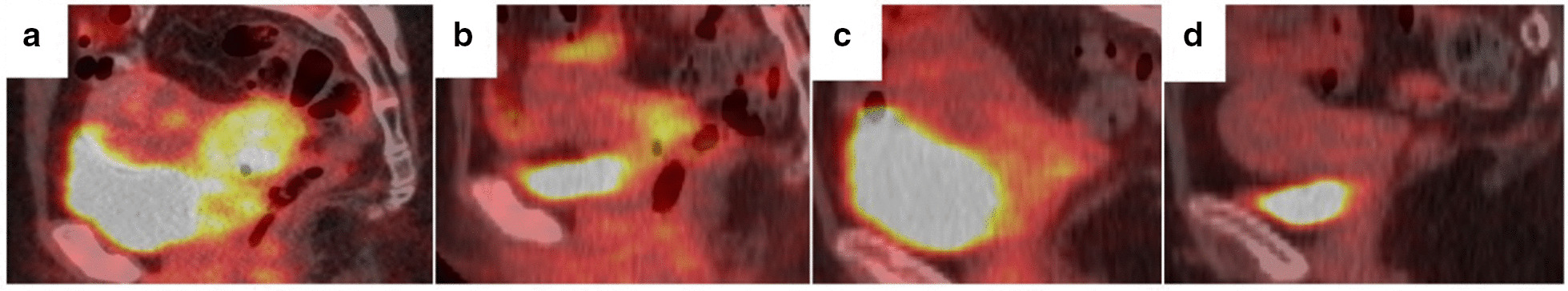
Dynamics of tumor by FDG-PET/CT. **a** Study at the onset of the disease; **b** study after course of induction therapy; **c** study after course of consolidation therapy; **d** study after course of specific therapy with Venetoclax and 5-azacitidine

Laboratory studies revealed the following values: hemoglobin 10.9 g/dL, platelet—177 × 10^9^/l, and white-cell count 3500 per cubic millimeter without pathological findings at leucogram. A bone marrow biopsy showed the presence of 16.2% positive MPO pathological blasts. The immunophenotypic analysis showed the expression of CD117, CD13, CD33, CD64, CD4, and MPO. Karyotype was normal—46, XX (20 metaphases were analyzed), while FISH demonstrated inversion 3 (identified the separation of the fusion signal from region 3q26 at 76 out of 200 analyzed interphase nuclei). A mutation in NPM1B was detected by quantitative PCR (737.8% copies). Lumbar puncture with prophylactic intrathecal chemotherapy was made and there was no evidence of CNS involvement. A pelvic magnetic resonance imaging (MRI) with contrast revealed an intrapelvic lymph nodes lesion and 43 × 75 × 76 mm tumor originating from the uterine cervix with invasion of the uterine corpus, parametrium, and pelvic peritoneum. A FDG-PET/CT showed a 71 × 48 mm lesion at the cervix with a standard uptake value maximum (SUVlbm max) of 5.5 (Fig. 2). Other hypermetabolic focuses were noted at intrapelvic lymphatic nodes: along the common iliac vessels (up to 8 mm with SUVlbm max = 2.16 on the right and up to 7 mm with SUVlbm max = 2.2 on the left), and the external iliac vessels (up to 15 × 11 mm with SUVlbm max = 2.6 on the right and 19 × 14 mm with SUVlbm max = 2.9 on the left). These findings were compatible with the diagnosis of MS involving the cervix and corpus of the uterus, parametrium, pelvic lymph nodes, and bone marrow.

The patient received AML type induction chemotherapy with cytosine arabinoside (200 mg/m^2^/day for 7 days) and daunorubicin (60 mg/m^2^ for 3 days). The course was complicated by febrile neutropenia and probable invasive mycosis which was successfully treated. The patient achieved complete clinical and hematologic remission (platelet—373 × 10^9^/l, absolute neutrophil count—1360 per cubic millimeter, bone marrow blasts—1%, independence of red cell transfusions) and partial metabolic response (decrease of tumor mass by 80.3%). The consolidation chemotherapy included high-dose cytosine arabinoside (3 g/m^2^/day). The control PET/CT showed the decrease in tumor mass (by 93% comparing to the initial size), and we were unable to achieve complete remission due to extramedullary lesion. Considering the data available in the literature about the successful use of BCL-2 inhibitors in combination with hypomethylating agents in AML, including extramedullary lesions [13, 14, 18], the patient underwent a course of chemotherapy with Venetoclax and 5-azacitidine (75 mg/m^2^). Post-chemotherapy examination demonstrated that the patient achieved complete MRD-negative remission (Fig. 2).

The patient underwent allogeneic hematopoietic stem cell transplantation from human leukocyte antigens (HLA)-matched related donor (sister) with myeloablative conditioning regimen (Fludarabine 150 mg/m^2^, Busulfan 12 mg/kg). Posttransplant period was complicated by sepsis, associated with Klebsiella pneumoniae, and systemic invasive mycosis involving the liver, spleen, and lungs, which were successfully treated. The patient achieved complete donor chimerism (100%) in 6 weeks after bone marrow transplantation. There was no evidence of graft-versus-host disease. The patient remains in complete MRD-negative and PET-negative remission for 4 months after bone marrow transplantation.

## Discussion

MS involving the female reproductive system occurs rather rarely. We were able to identify 57 cases of leukemic cervical tumors in published articles from 2002 to 2019 (Table 1). Patients often complain of vaginal bleeding at the onset of disease [6, 7], it was the first symptom in the above case. FDG-PET/CT appears to be the best imaging option to assess the presence of extramedullary AML and effectively used to both search for other possible lesions and monitoring the response to therapy [19–21]. MS may often be misdiagnosed for malignant lymphoma, small cell carcinoma, and undifferentiated tumor, so performing immunohistochemistry study with epithelial, neuroendocrine, myeloid, and lymphoid markers it is highly recommended [1, 2, 8, 21, 22]. Determining the markers, characteristic for myeloid cells (MPO, LCA) and the absence of lymphoid (CD20, CD10, CD3, CD30, CD7), epithelial (MCK, CK7), neuroendocrine (chromogranin A, synaptophysin), and other (ALK, desmin, Fli-1, S-100) markers allowed us to decide on the diagnosis. Additionally, the high expression of BCL-2 was found in the biopsy sampling.

**Table 1:** Literature review of myeloid sarcoma involving the gynecologic tract

No	Authors, country, year of study	Number of cases (n)	Affected region	Concurrent AML	Treatment	% (n)	Duration of observation (months)
1 [6]	B. Pathak et al., Canada, 2005	25	Cervix—76% (n = 19) Uterine corpus—8% (n = 2) External genital organs—4% (n = 1) ≥ 2 gynecologic sites—4% (n = 1) Not stated—8% (n = 2)	48% (n = 12)	CT	28 (7)	7.7
					RT	8 (2)	5
					SI	8 (2)	3
					CT + RT	24 (6)	13.75
					CT + SI	8 (2)	20.5
					RT + SI	4 (1)	1.75
					Without treatment	16 (4)	< 1
2 [40]	R.M. Kahn et al., USA, 2019	1	≥ 2 gynecologic sites (uterine corpus, fallopian tubes, left ovary)	Yes	CT + SI		3
3 [12]	G.Modi et al., India, 2004	1	External genital organs (vagina)	No	Hypomethylating therapy with decitabine		> 4
4 [41]	J.-A. Hernández et al., Spain, 2002	2	Cervix, left mesosalpinx, ovaries	100% (n = 2)	CT + SI + autoBMT		10
			External genital organs (vagina)		CT + autoBMT		10
5 [42]	M. Ucar, M. Guryildirim, Turkey, 2014	1	Breast, cervix, uterus	Yes	CT		> 2
6 [43]	M.G. Garcia et al., USA, Spain, 2006	11	Cervix—27.3% (n = 3) Ovary—18.2% (n = 2) Uterine corpus—9% (n = 1) External genital organs—9% (n = 1) ≥ 2 gynecologic sites—36.5% (n = 4)	36.5% (n = 4)	CT	64 (7)	40
					RT	9 (1)	5
					CT + alloBMT	9 (1)	> 6
					SI	73 (8)	-
		9	Cervix—11% (n = 1) Ovary—89% (n = 8)	22% (n = 2)	CT	33 (3)	15
					CT + alloBMT	11 (1)	10
7 [44]	S.C.H. Kim et al., France, 2010	1	Cervix	No	CT + RT		> 72
8 [45]	H. Gill et al., China, 2012	1	Cervix, left pelvic cavity	No	CT		16
9 [7]	W. Gui et al., China, 2019	2	Cervix, external genital organs (vagina), intrapelvic lymph nodes	No	SI + CT		21
			Cervix, uterus, intrapelvic lymph nodes	Yes	CT		26
10 [46]	A.S. Weingertner et al., France, 2009	1	Cervix, intrapelvic lymph nodes	Yes	CT		> 3
11 [47]	Y. Yu et al., China, 2015	1	External genital organs (vulva, vagina), cervix	No	CT		4
12 [48]	H. Bao et al., China, 2019	1	External genital organs	Yes	SI + CT + alloBMT		> 6

*CT* chemotherapy, *RT* radiotherapy, *SI* surgical intervention, *autoBMT* autologous bone marrow transplantation, *alloBMT* allogeneic bone marrow transplantation

Certain AML genetic abnormalities, like t(8;21), inv(16) and t(9;11), mutations in NPM1, NRAS, and DNMT3A, are associated with a higher incidence of extramedullary disease [1, 3, 5, 23]. Apart from a mutation in NPM1B, we also identified inversion of chromosome 3, which is strongly associated with poor prognosis in AML patients [24]. No association of chromosome 3 inversion with the extramedullary lesion was found in the literature.

The genetic assessment of extramedullary tumor is indeed, especially in a case of isolated disease [21], although these data are lacking in our case as in the majority of others, presented in Table 1.

MS is a systemic disease and AML-type regimens are most often recommended as initial therapy as stated in National Comprehensive Cancer Network and European Leukemia Net (2017) guidelines [19, 24]. Earlier studies suggested, MS had had the worse prognosis, in comparison to AML without extramedullary disease [25, 26]. Currently, the majority of authors consider that extramedullary AML is not per se associated with poor prognosis [16, 21].

Standard cytarabine-containing regimens, such as «7 + 3», HDAC (high doses of cytarabine), are used for eligible patients [15, 16, 19, 24]. This regimen was applied to our patient. Bone marrow MRD-negative response was obtained, that looked surprising: usually patients with chromosome 3 abnormality are resistant to chemotherapy: CR—31%, 5-year OS—5.7% ± 3%; EFS—0%; RFS-4.3% ± 4% [27]. Nevertheless, failure of metabolic response in extramedullary disease was registered according to the control PET/CT. Reasons for this discordant are unclear. Cunningham et al. attribute them to gene or microenvironmental deregulations [20, 28, 29], but that is continued to be studied.

In patients with chemotherapy resistant MS, radiation therapy is standardly recommended as second line of treatment [1, 11, 15, 16, 21]. Delay in systemic therapy often results to early relapse in patient with chromosome 3 inversion [27, 30, 31]. Thus, we thought about alternative regimens that could be used instead of or as an addition to irradiation.

Recently registered medicines such as anti-CD33 monoclonal antibody [32, 33], tyrosine kinase inhibitors (for FIP1L1-PDGFR [34, 35], and FLT3-ITD [36]), and DNA methyltransferase inhibitors [12] offer the possibility to continue systemic therapy for patients with MS.

The expression of CD33 was not studied in the biopsied tissue, targets for FLT-3 inhibitors were lacking in our patient. Targeting of BCL-2, involving mitochondrial pathway of apoptosis, has emerged as an efficacious and well-tolerated clinical strategy. It is based on founded on myeloblast function rather than on genetics [37, 38]. Regarding MS, overexpression of BCL-2 was found by Wang et al. [39]. Worth mentioning, that the tumor tissue of our patient was revealed to hyperexpression of BCL-2.

Combination of Venetoclax with 5-azacytidine was described as a very promising regimen in several case reports as well as the retrospective study of the small number of patients [13, 14]. This therapeutic option resulted in complete metabolic response and MRD-negative complete remission. Regimen was well tolerable without unusual toxicities. The prompt response of the extramedullary tumor to the combination of Venetoclax with 5-azacytidine allowed us to proceed to allogeneic hematopoietic SCT.

Allogeneic SCT is believed to improve OS in patients both with isolated MS and concurrent AML [8, 17]. Transplantation is usually recommended as consolidative strategy of extramedullary AML based on the disease risk (by cytogenetics or molecular data) and in case of resistance [16, 21]. Patient has been in complete remission until the present.

We report for the first-time successful treatment of uterine cervix MS with Venetoclax-based regimen is described. Of note, this is the first description MS of the uterine cervix with concurrent bone marrow involvement with chromosome 3 inversion.

## Conclusion

MS of the gynecologic tract is a very rare and often misdiagnosed hematologic malignancy, whose diagnostics and therapeutic aspects remain a subject of discussion. MS treatment standard is AML-type chemotherapy. Postremission therapy is dependent upon a number of factors: extent of disease, risk profile, and performance status. The use of Venetoclax with hypomethylating agent has shown good results in our clinical case. Adding this combination to standard treatment regimens in patients with extramedullary AML might become a promising clinical option. The pre-transplant therapy allowed us to perform allogeneic bone marrow transplantation with the deepest response possible: complete PET-negative and MRD-negative remission of the disease.

Hematopoietic stem-cell transplantation remains an important therapeutic option.

## Data Availability

All data related to this case report are available from the corresponding author on reasonable request.

## Abbreviations

AML: Acute myeloid leukemia
BCL-2: B-cell lymphoma 2
CD: Cluster of differentiation
CNS: Central nervous system
CR: Complete remission
DNA: Deoxyribonucleic acid
ECOG: Eastern Cooperative Oncology Group
EFS: Event-free survival
FDG-PET/CT: Fluorodeoxyglucose positron emission tomography/computed tomography
FISH: Fluorescence in situ hybridization
FLT3: FMS-like tyrosine kinase 3
HLA: Human leukocyte antigens
LCA: Leucocyte common antigen
MCK: Multi-cytokeratin
MPO: Myeloperoxidase
MRD: Minimal residual disease
MRI: Magnetic resonance imaging
MS: Myeloid sarcoma
NPM1B: Nucleophosmin 1B
OS: Overall survival
PCR: Polymerase chain reaction
FDG-PET/CT: Positron emission tomography–computed tomography
RFS: Relapse-free survival
SCT: Stem cell transplantation
SUVlbm max: Standard uptake value maximum
